# Influence of Cardiometabolic Disease on Macular Structural Changes After Uncomplicated Phacoemulsification

**DOI:** 10.3390/jcm15166163

**Published:** 2026-08-08

**Authors:** Maria-Emilia Cerghedean-Florea, Cosmin Adrian Teodoru, Horațiu Dura, Adrian Hașegan, Adrian Boicean, Paul Șiancu, Denisa Tănăsescu, Mihaela Laura Vică, Horia Stanca, Tudor Călinici, Valeria Coviltir, Ciprian Tănăsescu

**Affiliations:** 1 Faculty of Medicine, “Lucian Blaga” University of Sibiu, 550024 Sibiu, Romania; mariaemilia.florea@ulbsibiu.ro (M.-E.C.-F.); tanasescuciprian@yahoo.fr (C.T.); 2 Department of Cellular and Molecular Biology, “Iuliu Haţieganu” University of Medicine and Pharmacy, 400012 Cluj-Napoca, Romania; 3 Institute of Legal Medicine, 400006 Cluj-Napoca, Romania; 4 Department of Ophthalmology, “Carol Davila” University of Medicine and Pharmacy, 050474 Bucharest, Romania; horia.stanca@umfcd.ro; 5 Department of Medical informatics and Biostatistics, “Iuliu Haţieganu” University of Medicine and Pharmacy, 400012 Cluj-Napoca, Romania; 6 Ophthalmology Discipline, “Carol Davila” University of Medicine and Pharmacy, 8 Eroii Sanitari Blvd, 050474 Bucharest, Romania; 7 Department of Ophthalmology, Clinical Institute of Ophthalmological Emergencies “Prof. Dr. Mircea Olteanu”, 010464 Bucharest, Romania

**Keywords:** phacoemulsification, optical coherence tomography, macular thickness, macular volume, cardiometabolic disease, retinal remodeling

## Abstract

**Background/Objectives**: To evaluate macular structural changes after uncomplicated phacoemulsification using optical coherence tomography (OCT) and to investigate whether cardiometabolic disease influences the early postoperative retinal response. **Methods**: This retrospective observational study included 111 eyes from 111 patients undergoing uncomplicated phacoemulsification with intraocular lens implantation. Macular OCT parameters were evaluated preoperatively and at postoperative days 1, 7, and 30. Patients were stratified according to the presence of cardiometabolic disease, and retinal changes were compared between groups. **Results**: Exploratory visit-specific comparisons produced nominal differences in average retinal thickness and macular volume at several assessments; however, none remained statistically significant after Holm adjustment across the 16 parameter-by-visit comparisons. The adjusted models provided no statistically significant evidence that postoperative changes from baseline differed according to cardiometabolic status. Model-derived interaction contrasts at days 1, 7, and 30 were small and their 95% confidence intervals included zero for all OCT outcomes. Accordingly, no statistically significant differences in postoperative change from baseline were detected between groups; equivalence was not assessed. **Conclusions**: Although nominal differences were observed in exploratory visit-specific comparisons, none remained statistically significant after multiplicity adjustment. Furthermore, the adjusted analyses did not detect statistically significant differences in postoperative change from baseline according to cardiometabolic status after adjustment for baseline retinal structure and relevant demographic and surgical confounders. No statistically significant evidence of different postoperative OCT trajectories was detected between groups; equivalence was not assessed.

## 1. Introduction

Cataract surgery is the most common ophthalmic procedure and remains the only effective option for treating cataracts. In developed countries, phacoemulsification is the gold standard due to its small incision, rapid recovery, reduced postoperative astigmatism, and short healing time. The increase in the number of cataract surgeries is correlated with rising life expectancy and the prevalence of the disease [1,2,3]. During phacoemulsification, various procedural parameters can affect the structure of ocular tissues. In particular, ultrasonic energy and fluid flow generate mechanical effects that can cause inflammation, compression, and local tissue hypoxia [4].

Cataract surgery may induce subtle structural retinal changes, even after uncomplicated procedures. Because these changes may be more relevant in patients whose retinal microvasculature is already affected by systemic conditions, the question arises whether patients with cardiometabolic risk factors present a different retinal response to surgery. Like other organs, the retina is susceptible to endothelial dysfunction caused by insufficient perfusion and capillary atrophy, changes in torsional force due to systemic hypertension, neurovascular decoupling resulting from reduced oxygen and nutrient supply, as well as oxidative stress and inflammation secondary to ischemia [5,6,7,8].

Cardiometabolic disorders, including hypertension, diabetes mellitus, dyslipidemia, ischemic heart disease, heart failure, and atrial fibrillation, have been associated with structural changes in the retina and choroid. Although these conditions differ in their clinical manifestations, they are all associated with systemic vascular dysfunction and retinal microvascular alterations. Reduced choroidal thickness, macular thinning, and retinal microvascular remodeling have been reported across these conditions, indicating that OCT-derived retinal biomarkers may provide noninvasive indicators of systemic microvascular impairment and cardiometabolic risk [9,10].

Optical Coherence Tomography (OCT) imaging allows for microscopic evaluation of the retina. Similar to the principle behind ultrasound, OCT emits light and measures variations in the light reflected by the retina, enabling the creation of a cross-sectional image to visualize, measure, and detect morphological changes in the distinct layers of the retina [5]. Assessment of these parameters via OCT provides a non-invasive and accurate method for detecting retinal structural changes associated with cardiovascular and metabolic diseases. Systemic factors such as hypertension, diabetes, hypercholesterolemia, and renal insufficiency influence the thickness of the choroid and the retinal nerve fiber layer [11,12,13,14,15].

The primary objective of this study was to evaluate postoperative macular structural changes after uncomplicated phacoemulsification using spectral-domain OCT. A second objective was to investigate whether cardiovascular and metabolic risk factors, together with intraoperative phacoemulsification parameters, influence postoperative retinal dynamics. The study aimed to identify subtle retinal changes and their correlations with systemic comorbidities, providing further insight into the impact of these risk factors on retinal structure, even in the absence of clinically evident retinal disease.

## 2. Materials and Methods

### 2.1. Study Design

This retrospective observational study was conducted over a period of 6 months at Arcada Clinic, Sibiu, Romania, and included 111 eyes from 111 patients who underwent uncomplicated phacoemulsification cataract surgery with intraocular lens implantation. Because this was a retrospective study, no a priori sample-size calculation was performed. All patients who met the eligibility criteria and had complete OCT measurements during the predefined study period were included. Informed consent was obtained from all subjects involved in the study. The study was conducted in accordance with the Declaration of Helsinki and by the ethics committee of the “Lucian Blaga” University in Sibiu (No. 9, date of approval: 29 July 2022).

Pre- and postoperative evaluations included measurements of visual acuity and intraocular pressure, examination of the anterior segment and the fundus, as well as structural assessment of the retina using optical coherence tomography (OCT) to evaluate macular parameters. Visual acuity was measured preoperatively and postoperative (day 30). Systemic cardiovascular and metabolic risk factors, including hypertension, type 2 diabetes mellitus, dyslipidemia, ischemic heart disease, heart failure, and atrial fibrillation, were recorded for all cases. Patients with at least one of these conditions were classified as having cardiometabolic disease, whereas those without any of these conditions constituted the comparison group. Intraoperative parameters such as ultrasound time, cumulative dissipated energy (CDE), torsional time, average phaco power, aspiration time, and estimated fluid usage were also analyzed.

### 2.2. Inclusion and Exclusion Criteria

The study included patients diagnosed with cataracts who were scheduled for phacoemulsification with intraocular lens implantation, whose surgery was performed without intraoperative complications, and for whom complete OCT evaluations were available, performed preoperatively and postoperatively at the specified time points (day 1, day 7, and day 30). Only patients who signed the informed consent form and who could be monitored throughout the follow-up period were included.

Patients with ocular conditions that could have influenced the analyzed retinal parameters or the quality of the OCT examination were excluded, such as glaucoma, significant corneal opacities, ocular trauma, active ocular infections, intraoperative or postoperative complications, as well as cases in which the surgical procedure required significant deviations from the standard technique. Patients with OCT images of inadequate quality, affected by motion artifacts, blinking, or incorrect centering, as well as cases with incomplete data at postoperative follow-ups, were also excluded. Only one eye per patient was included in the analysis. If both eyes underwent cataract surgery during the study period, only the first operated eye that met the inclusion and exclusion criteria was included.

### 2.3. Surgical Technique

All procedures were carried out by the same surgeon using an Infiniti^®^ Vision System with an OZil^®^ torsional handpiece (Alcon Laboratories Inc., Fort Worth, TX, USA). Pupillary dilation was achieved with topical tropicamide 1% and phenylephrine 10% eye drops, and topical anesthesia was obtained with oxybuprocaine 0.4% eye drops.

Access to the anterior chamber was gained through a 2.2 mm superior corneal incision, complemented by two lateral paracenteses. An ophthalmic viscosurgical device was instilled to maintain anterior chamber stability and protect the corneal endothelium. A continuous curvilinear capsulorhexis was fashioned with capsulotomy forceps, after which hydrodissection and hydrodelineation were performed to allow free rotation of the nucleus. Nuclear disassembly was accomplished by divide-and-conquer and stop-and-chop techniques, depending on lens density.

Once phacoemulsification was complete, a posterior chamber intraocular lens was implanted in the capsular bag. Lens selection was individualized according to each patient’s refractive requirements.

### 2.4. OCT Measurements

Retinal structural assessment was performed using spectral-domain optical coherence tomography (OCT) with a REVO 80 device (Optopol Technology, Zawiercie, Poland; REVO NX software version 11.5.1). Macular imaging was acquired using the standard 3D 7 × 7 mm scan protocol preoperatively and at postoperative days 1, 7, and 30. The macular parameters provided by the device’s automated segmentation algorithm, namely minimum foveal thickness, central sector thickness, average retinal thickness, and macular volume, were analyzed. Measurements were obtained from the automatically generated ETDRS retinal thickness map (1, 3, and 6 mm). Only scans with a Quality Index (QI) of at least 7, correct foveal centration, and without significant motion, blinking, or segmentation artifacts were included in the analysis. All examinations were performed according to the same acquisition protocol and at the same postoperative follow-up intervals.

### 2.5. Statistical Analysis

Data were collected in Microsoft Excel (Microsoft Corp., Redmond, WA, USA) and analyzed using jamovi version 2.7 (The jamovi project, Sydney, Australia). Categorical variables are presented as frequencies and percentages. Continuous variables are reported as mean ± standard deviation when their distributions were consistent with normality and as median (interquartile range) when normality was not supported. Means and medians were not reported simultaneously unless both were required for a specifically justified descriptive purpose. For inferential comparisons, the statistical test, effect-estimate direction, 95% confidence interval, and exact *p*-value are reported whenever applicable. *p*-values below 0.001 are reported as *p* < 0.001. Decimal points are used throughout the manuscript.

Average phaco power was recorded as a proportion and converted to a percentage by multiplying the recorded value by 100.

Normality was assessed using the Shapiro–Wilk test both in the overall cohort and separately within each comparison group. For between-group analyses, variables were analyzed using the Mann–Whitney U test and presented as median with interquartile range when the distribution in either group deviated significantly from normality. Independent-samples *t*-tests and mean ± standard deviation were used only when normality was supported in both groups.

When the Friedman test indicated a significant overall time effect, post hoc pairwise comparisons were performed using the Durbin–Conover procedure. To control the family-wise error rate, the six pairwise comparisons within each OCT parameter were adjusted using the Holm step-down procedure.

The visit-specific between-group comparisons across the four OCT parameters and four assessment times were considered exploratory. To account for the 16 comparisons, Holm-adjusted *p*-values were calculated in addition to the nominal *p*-values. Statistical significance for these exploratory comparisons was evaluated using the Holm-adjusted values. The mixed-effects time-by-group interaction remained the primary inferential test of whether longitudinal OCT trajectories differed according to cardiometabolic status.

For comparisons involving non-normally distributed variables, the between-group effect estimate was expressed as the rank-biserial correlation, which is directly related to the Mann–Whitney U statistic. Rank-biserial correlations were calculated as the group without cardiometabolic disease relative to the group with cardiometabolic disease and are reported with large-sample 95% confidence intervals based on the variance of the pairwise comparison probabilities, with ties assigned half weight.

Repeated OCT measurements were analyzed using separate linear mixed-effects models for minimum foveal thickness, central sector thickness, average retinal thickness, and macular volume. Each model included a patient-specific random intercept to account for within-patient correlation. Fixed effects included assessment time as a categorical variable (preoperative, postoperative day 1, day 7, and day 30), cardiometabolic disease status, and the interaction between assessment time and cardiometabolic disease status. Age, sex, cumulative dissipated energy, and ultrasound time were included as covariates. The preoperative OCT measurement was included as the first repeated outcome. Because the preoperative OCT measurement was modeled as part of the four-visit outcome trajectory, it was not simultaneously entered as a covariate in the primary model. The cardiometabolic-status main effect estimated the adjusted baseline group difference, whereas the time-by-group interaction evaluated whether subsequent changes relative to baseline differed between groups. As a sensitivity analysis, the models were restricted to postoperative days 1, 7, and 30, and the corresponding preoperative OCT value was included as an additional covariate. Raw changes from baseline to postoperative day 30 were summarized as a secondary descriptive analysis to facilitate clinical interpretation. Because change-score comparisons may be affected by baseline variability and regression to the mean, they were not used as the primary test of the study hypothesis. Primary inference was based on the mixed-effects models incorporating all visits, together with sensitivity models of the postoperative measurements adjusted for the corresponding baseline OCT value.

The overall time-by-group interaction was the primary test of whether the pattern of OCT change differed between patients with and without cardiometabolic disease. Models were estimated using maximum likelihood. Time-by-group interactions were evaluated using likelihood-ratio tests and are reported as χ^2^ statistics with the corresponding degrees of freedom and *p*-values. Collinearity between covariates was evaluated using variance inflation factors. A two-tailed *p*-value below 0.05 was considered statistically significant.

To characterize the precision provided by the available group sizes, minimum detectable between-group differences were calculated for the baseline-to-day-30 change scores using a two-sided significance level of 0.05 and 80% statistical power. Calculations were based on the observed group sizes and pooled standard deviations. Retrospective observed power was not calculated because it provides no additional information beyond the estimated effects, confidence intervals, and *p*-values.

Exploratory sensitivity analyses were performed to investigate the heterogeneity of the cardiometabolic group. Postoperative OCT trajectories were compared between patients with and without type 2 diabetes mellitus in the entire cohort and, within the cardiometabolic group, between patients with hypertension alone and those with other cardiometabolic profiles. Separate linear mixed-effects models were fitted for each OCT parameter using the three postoperative assessments. The models included assessment time, subgroup status, and their interaction, with a patient-specific random intercept and adjustment for the corresponding baseline OCT value, age, sex, cumulative dissipated energy, and ultrasound time. Because eight time-by-subgroup interaction tests were performed across the two sensitivity analyses and four OCT parameters, Holm-adjusted *p*-values were calculated. These analyses were considered exploratory.

Model-derived time-by-cardiometabolic-status interaction contrasts were estimated at postoperative days 1, 7, and 30 relatives to the preoperative assessment and are reported with 95% confidence intervals.

Artificial intelligence tools were used exclusively for language editing and paraphrasing of the manuscript (ChatGPT, OpenAI, San Francisco, CA, USA; GPT-5.2).

## 3. Results

### 3.1. Demographic Characteristics, Clinical Features and Comorbidities of the Study Population

A total of 111 eyes from 111 patients meeting the inclusion criteria were enrolled in the present study. The demographic characteristics, clinical features and associated comorbidities of the study population are presented in Table 1.

Of the patients included, 64 (57.7%) were female and 47 (42.3%) were male. The median age was 71 years (IQR: 11 years). Most patients came from urban areas, 85 cases (76.6%), compared with 26 cases (23.4%) from rural areas. The distribution of procedures according to the operated eye was similar, with a slight predominance of the right eye, 61 cases (55.0%), compared with the left eye, 50 cases (45.0%).

Regarding comorbidities, arterial hypertension was the most frequently encountered associated condition, present in 67 patients (60.4%). Type 2 diabetes mellitus was identified in 25 patients (22.5%), dyslipidemia in 19 patients (17.1%), and ischemic heart disease in 13 patients (11.7%). Heart failure and atrial fibrillation were present in a small number of patients. Within the 70-patient cardiometabolic group, hypertension was present in 67 patients (95.7%), type 2 diabetes mellitus in 25 (35.7%), dyslipidemia in 19 (27.1%), ischemic heart disease in 13 (18.6%), heart failure in 5 (7.1%), and atrial fibrillation in 2 (2.9%). Twenty-four patients (34.3%) had one recorded cardiometabolic condition, 33 (47.1%) had two, 11 (15.7%) had three, and 2 (2.9%) had four conditions.

### 3.2. Preoperative and Postoperative Visual Acuity

Visual acuity was assessed preoperatively and at postoperative (day 30) in all 111 patients. Preoperative and postoperative values, expressed in decimal notation, are presented in Table 2.

Preoperative and postoperative visual-acuity measurements were not normally distributed and are therefore presented as medians with interquartile ranges. Median visual acuity increased from 0.300 (IQR 0.400) preoperatively to 1.00 (IQR 0.100) postoperatively. The median paired improvement was 0.550 decimal units (95% CI 0.500 to 0.600; Wilcoxon signed-rank *p* < 0.001). No patient showed a decrease from the preoperative value.

### 3.3. Intraoperative Parameters of the Phacoemulsification Surgery

The intraoperative parameters recorded for the 111 patients are presented in Table 3. Cumulative dissipated energy (CDE) had a median value of 6.26%·s (IQR 7.99), while average phaco power had a median value of 27.1% (IQR 13.6%). The average phaco-power values were recorded in the database as proportions and were multiplied by 100 for presentation as percentages. Ultrasound time had a median of 45 s (IQR 38.0), and torsional time a median of 43 s (IQR: 39.0). Aspiration time showed a median value of 259 s (IQR 116), and the estimated volume of fluid used was 79 mL (IQR 26.5).

All intraoperative parameters showed significant deviations from a normal distribution on the Shapiro–Wilk test (all *p* < 0.05; Table 3); accordingly, values are reported preferentially as median and interquartile range.

Descriptive and unadjusted analyses are presented to characterize the observed OCT values and individual time-point comparisons. The mixed-effects model incorporating all four visits was considered the primary inferential analysis of whether longitudinal OCT changes differed between groups.

### 3.4. Changes in Macular Parameters Assessed by Optical Coherence Tomography (OCT)

Macular structural parameters were assessed preoperatively and at 1, 7 and 30 days after surgery. Longitudinal analysis revealed significant changes in all four macular parameters throughout the follow-up period (all *p* < 0.001). Overall results are presented in Table 4, and pairwise post hoc comparisons in Table 5.

Minimum foveal thickness showed a significant change over time (χ^2^ = 65.8; df = 3; *p* < 0.001). The median value increased from 201 μm preoperatively to 210 μm at 30 days postoperatively. Post hoc analysis revealed no significant change between the preoperative assessment and day 1 (*p* = 0.365), whereas values at day 7 and day 30 were significantly higher than baseline (both *p* < 0.001). A significant increase was also observed between day 7 and day 30 (*p* < 0.001).

Central sector thickness followed a similar pattern (χ^2^ = 116; df = 3; *p* < 0.001). Values remained stable on the first postoperative day (*p* = 0.185) and subsequently increased progressively at the day 7 and day 30 assessments. Compared with the preoperative examination, differences were significant at both day 7 and day 30 (both *p* < 0.001), and the increase between day 7 and day 30 remained statistically significant (*p* < 0.001).

Average retinal thickness showed a different pattern compared with the first two parameters (χ^2^ = 132; df = 3; *p* < 0.001). On the first postoperative day, a slight but statistically significant decrease was observed compared with the preoperative value (273 μm versus 272 μm, *p* = 0.020), followed by a progressive increase at the day 7 and day 30 assessments. At 30 days, the median value was significantly higher than the preoperative one (280 μm versus 273 μm, *p* < 0.001). The difference between day 7 and day 30 was also significant (*p* < 0.001).

Macular volume showed a temporal pattern similar to that of average retinal thickness (χ^2^ = 142; df = 3; *p* < 0.001). On the first postoperative day, a slight reduction was recorded compared with the preoperative value (7.70 mm^3^ versus 7.74 mm^3^, *p* = 0.008), followed by a progressive increase at subsequent assessments. At 30 days, macular volume was significantly higher than at the preoperative assessment (7.90 mm^3^ versus 7.74 mm^3^, *p* < 0.001). The difference between day 7 and day 30 remained statistically significant (*p* < 0.001).

### 3.5. The Influence of Cardiometabolic Pathology on Macular Parameters and Postoperative Response

Patients were divided into two groups according to the presence of cardiometabolic pathology: without cardiometabolic pathology (n = 41) and with cardiometabolic pathology (n = 70). Comparisons between the two groups were performed using the independent samples *t*-test or the Mann–Whitney U test, depending on the distribution of the variables.

#### 3.5.1. Comparative Characteristics of the Two Groups

The comparative characteristics of the two groups are presented in Table 6. Normality was reassessed separately within each group. Because at least one group showed a significant deviation from normality for each continuous variable, comparisons were performed using Mann–Whitney U tests and values are presented as median with interquartile range. Patients with cardiometabolic pathology were older than those without cardiometabolic pathology, with median ages of 73.0 and 68.0 years, respectively (*p* < 0.001). Ultrasound time was longer in the cardiometabolic group: 47.5 s (IQR 32.75) versus 39.0 s (IQR 35.0), corresponding to a rank-biserial correlation of −0.232 (95% CI −0.451 to −0.012; *p* = 0.042). Cumulative dissipated energy was numerically higher in the cardiometabolic group, but the difference did not reach statistical significance: 6.50%·s (IQR 7.67) versus 5.19%·s (IQR 5.99; *p* = 0.059).

No statistically significant between-group differences were detected in preoperative visual acuity (*p* = 0.988), postoperative visual acuity (*p* = 0.392), aspiration time (*p* = 0.737), or estimated fluid usage (*p* = 0.300). The categorical comparisons of sex, residence, and operated eye also showed no statistically significant differences between groups.

#### 3.5.2. Comparison of Macular OCT Parameters

The comparisons presented in this subsection are unadjusted and describe the observed absolute OCT values in the two groups.

No statistically significant differences were identified between the two groups for minimum foveal thickness and central sector thickness at any assessment time point (all *p* > 0.05).

In the unadjusted analyses, average retinal thickness was higher in patients without cardiometabolic pathology at each assessment. At the preoperative assessment, the mean between-group difference was 6.57 µm (95% CI 0.57 to 12.57; *p* = 0.032). The corresponding mean differences were 7.24 µm at day 1 (95% CI 1.59 to 12.89; *p* = 0.012) and 7.20 µm at day 7 (95% CI 1.32 to 13.07; *p* = 0.017). At day 30, the distribution in the cardiometabolic group deviated significantly from normality; therefore, values were summarized as medians and compared using the Mann–Whitney U test. Median average retinal thickness was 285 µm (IQR 20.0) in patients without cardiometabolic pathology and 277 µm (IQR 21.0) in patients with cardiometabolic pathology (U = 1006.5; *p* = 0.009).

Macular volume was also lower in the cardiometabolic group in the unadjusted analyses. At day 1, the mean difference was 0.174 mm^3^ (95% CI 0.011 to 0.337; *p* = 0.036), and at day 7 it was 0.174 mm^3^ (95% CI 0.009 to 0.340; *p* = 0.039). At day 30, macular volume deviated significantly from normality in the cardiometabolic group and was therefore analyzed using the Mann–Whitney U test. Median macular volume was 8.03 mm^3^ (IQR 0.600) in patients without cardiometabolic pathology and 7.81 mm^3^ (IQR 0.598) in patients with cardiometabolic pathology (U = 1002; *p* = 0.008). The preoperative difference in macular volume was not statistically significant. Although several nominal *p*-values were below 0.05, none of the 16 parameter-by-visit comparisons remained statistically significant after Holm adjustment (Table 7).

#### 3.5.3. Postoperative Changes in Macular Parameters

As a secondary descriptive analysis, changes from baseline to postoperative day 30 are presented in Table 8. The mean between-group differences, calculated as the change in patients with cardiometabolic disease minus the change in patients without cardiometabolic disease, were −0.06 µm for minimum foveal thickness (95% CI −4.74 to 4.62), 1.00 µm for central sector thickness (95% CI −2.97 to 4.97), −1.11 µm for average retinal thickness (95% CI −3.73 to 1.51), and −0.036 mm^3^ for macular volume (95% CI −0.102 to 0.030). These unadjusted change scores were not used as the primary inferential analysis because they do not account optimally for baseline differences and may be affected by regression to the mean. Primary interpretation was therefore based on the longitudinal mixed-effects models and the baseline-adjusted sensitivity analyses reported below.

Given the available group sizes of 70 and 41 patients, the minimum detectable between-group differences at 80% power and a two-sided α level of 0.05 were 6.68 µm for minimum foveal thickness, 5.66 µm for central sector thickness, 3.73 µm for average retinal thickness, and 0.094 mm^3^ for macular volume. Accordingly, the study had sufficient power to detect approximately moderate standardized effects but may not have detected smaller differences.

#### 3.5.4. Mixed-Effects Analysis of Longitudinal OCT Changes

To determine whether the complete pattern of OCT change differed according to cardiometabolic status, separate linear mixed-effects models were fitted for the four OCT outcomes across the preoperative, day 1, day 7, and day 30 assessments. Each model included a patient-specific random intercept and fixed effects for assessment time, cardiometabolic disease status, and their interaction, with adjustment for age, sex, cumulative dissipated energy, and ultrasound time.

No statistically significant overall interaction between cardiometabolic status and assessment time was identified for any OCT outcome (Table 9). The model-derived interaction contrasts at days 1, 7, and 30 were small, and all 95% confidence intervals included zero. Thus, the models did not detect statistically significant differences between groups in change from baseline at any postoperative assessment. The confidence intervals describe the precision of these estimates and should not be interpreted as demonstrating equivalence.

Minimum foveal thickness and central sector thickness remained relatively stable on postoperative day 1 and subsequently increased at days 7 and 30. Average retinal thickness and macular volume showed a small initial reduction followed by progressive increases. These temporal patterns were observed in both groups. The nonsignificant interaction terms indicate that no statistically significant differences in the early longitudinal OCT response were detected according to cardiometabolic status.

**Figure 1:**
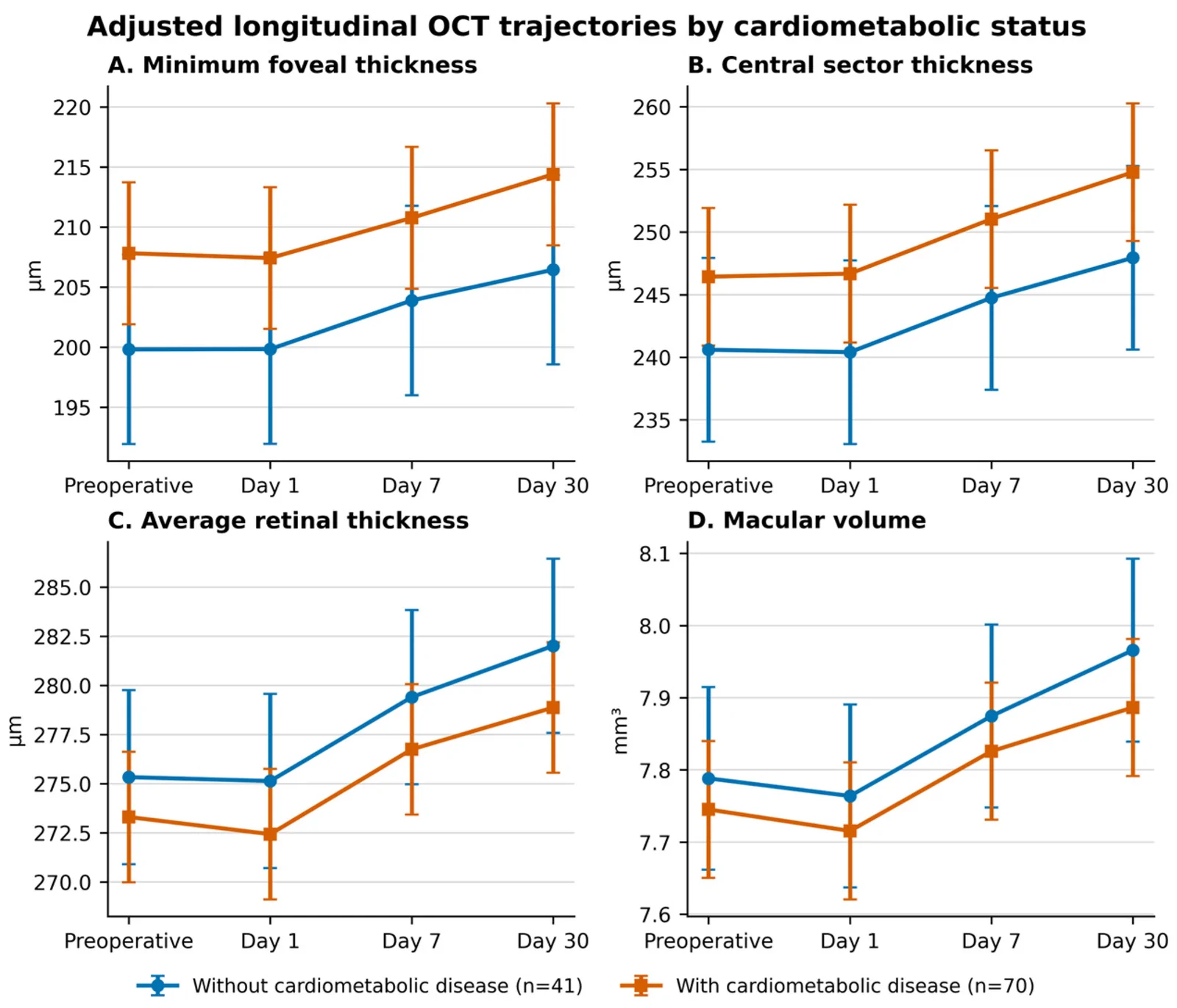
Adjusted longitudinal trajectories of OCT measurements.

Adjusted longitudinal trajectories of minimum foveal thickness (A), central sector thickness (B), average retinal thickness (C), and macular volume (D), stratified by cardiometabolic status are presented in Figure 1. Points represent model-adjusted estimated means and error bars represent 95% confidence intervals. Estimates were obtained from linear mixed-effects models incorporating all four assessments and adjusted for age, sex, cumulative dissipated energy, and ultrasound time. No statistically significant time-by-cardiometabolic-status interaction was identified for any OCT parameter.

The findings were confirmed in sensitivity analyses restricted to the three postoperative assessments and additionally adjusted for the corresponding baseline OCT value. No significant time-by-cardiometabolic-status interaction was observed for minimum foveal thickness (χ^2^ = 0.265; df = 2; *p* = 0.876), central sector thickness (χ^2^ = 0.166; df = 2; *p* = 0.920), average retinal thickness (χ^2^ = 0.204; df = 2; *p* = 0.903), or macular volume (χ^2^ = 1.470; df = 2; *p* = 0.479). Thus, adjustment for individual baseline OCT values did not provide statistically significant evidence of different postoperative trajectories between groups. These analyses were not designed to establish equivalence.

#### 3.5.5. Sensitivity Analyses

Exploratory subgroup sensitivity analyses were conducted to assess whether the heterogeneity of the cardiometabolic group influenced the findings. In the full cohort, 25 patients had type 2 diabetes mellitus and 86 did not. The baseline-adjusted postoperative mixed models did not identify nominally significant time-by-diabetes interactions for minimum foveal thickness (χ^2^ = 1.475; df = 2; *p* = 0.478), central sector thickness (χ^2^ = 3.956; df = 2; *p* = 0.138), or macular volume (χ^2^ = 5.050; df = 2; *p* = 0.080). A nominal interaction was observed for average retinal thickness (χ^2^ = 8.079; df = 2; *p* = 0.018), but this did not remain statistically significant after Holm adjustment across the eight exploratory interaction tests (adjusted *p* = 0.141).

Within the cardiometabolic group, 21 patients had hypertension alone and 49 had other cardiometabolic profiles. No time-by-subgroup interaction was detected for minimum foveal thickness (χ^2^ = 0.342; df = 2; *p* = 0.843), central sector thickness (χ^2^ = 0.928; df = 2; *p* = 0.629), average retinal thickness (χ^2^ = 1.405; df = 2; *p* = 0.495), or macular volume (χ^2^ = 1.779; df = 2; *p* = 0.411). None of these interactions remained significant after Holm adjustment. These subgroup analyses were exploratory and had limited precision because of the relatively small subgroup sizes.

Overall, none of the eight exploratory time-by-subgroup interaction tests remained statistically significant after Holm correction, providing no multiplicity-adjusted evidence that postoperative OCT trajectories differed according to diabetes status or between hypertension alone and other cardiometabolic profiles.

## 4. Discussion

The present study included 111 patients who underwent uncomplicated phacoemulsification and were evaluated for preoperative and postoperative macular structural changes. The demographic characteristics of the cohort were consistent with those expected for this patient population. The relatively advanced mean age reflects the predominantly age-related nature of cataract, the prevalence of which increases substantially after 60 years of age, affecting more than 50% of individuals within this age group [16,17]. With respect to sex distribution, the cohort demonstrated a slight female predominance. Although large scale meta-analyses have not identified a significant difference in cataract prevalence between women and men [16], several studies have suggested that female sex may represent a risk factor. This association has been attributed to age-related hormonal changes, cumulative lifetime exposure to environmental risk factors, and genetic susceptibility [18,19,20,21].

From the perspective of systemic comorbidities, arterial hypertension was the most prevalent associated condition, affecting the majority of patients, followed by type 2 diabetes mellitus and dyslipidemia. This frequent coexistence of vascular and metabolic risk factors among patients with cataract is well supported by recent evidence [22,23,24,25,26,27,28]. A large meta-analysis demonstrated that metabolic syndrome is associated with a significantly increased risk of cataract, approximately 28% higher than in individuals without metabolic syndrome, with a stronger association observed in older adults [29,30,31]. The high prevalence of these comorbidities within the study cohort enabled the identification of a cardiometabolic subgroup, which was analyzed separately to evaluate the potential influence of cardiometabolic disease on postoperative macular structural parameters.

Improvement in visual acuity was observed throughout the study cohort, with all patients demonstrating better postoperative visual acuity than at baseline and no cases of postoperative visual deterioration. This functional improvement confirms the effectiveness of phacoemulsification with intraocular lens implantation in the treatment of cataract and is consistent with the established role of this procedure as one of the safest and most effective surgical interventions in ophthalmology [32,33]. Notably, the universal improvement in visual acuity, despite the structural macular changes documented in the following sections, indicates that these anatomical alterations did not translate into measurable functional impairment. This dissociation suggests that the postoperative retinal alterations detected by OCT were subclinical.

Intraoperative parameters were recorded to explore potential determinants of postoperative macular changes. Cumulative dissipated energy and ultrasound time reflect the amount of ultrasonic energy delivered during surgery and, consequently, the extent of surgical tissue stress. Greater ultrasonic energy exposure may induce a more pronounced inflammatory response, resulting in increased postoperative macular thickening. This mechanism is particularly relevant when comparing patients with and without cardiometabolic disease. Patients with cardiometabolic disease had a significantly longer ultrasound time, whereas cumulative dissipated energy was only numerically higher and did not differ significantly between groups. These findings supported adjustment for both surgical parameters when evaluating whether postoperative retinal responses differed according to cardiometabolic status. All procedures were performed by a single surgeon using the same torsional phacoemulsification platform, thereby ensuring procedural standardization and minimizing variability related to surgical technique or operator experience.

Macular structural parameters were evaluated preoperatively and at 1, 7, and 30 days after surgery to characterize the retinal response to phacoemulsification. The four OCT-derived parameters, minimum foveal thickness, central sector thickness, average retinal thickness, and macular volume, all demonstrated a significant increase throughout the follow-up period. These findings are consistent with the well-documented phenomenon of postoperative macular thickening following uncomplicated phacoemulsification, even in the absence of intraoperative or postoperative complications [34,35,36,37].

The present results confirm that phacoemulsification induces measurable structural changes within the macula that can be detected by OCT. The temporal profile showed a heterogeneous pattern of change across the evaluated macular parameters. Minimum foveal thickness and central sector thickness remained relatively stable on the first postoperative day, whereas significant changes involving the entire macular area became evident at the 7- and 30-day evaluations. This pattern suggests that the parafoveal and peripheral macular regions are affected earlier than the central fovea. These findings are consistent with those reported by Gharbiya et al., who described early postoperative thickening in the parafoveal region, while central foveal thickness increased at a later stage [35]. A similar regional distribution has been reported by other authors, who observed more pronounced thickening within the 3 mm and 6 mm macular subfields [34].

Another important finding was the continuous increase in all macular parameters up to postoperative day 30, with no evidence of a plateau during the follow up period. This observation is in agreement with previous studies reporting that postoperative macular thickening typically reaches its maximum between 1 and 3 months after surgery, while subtle thickening in the peripheral macular regions may persist for up to 6 months following uncomplicated phacoemulsification [35,36].

Exploratory visit-specific analyses identified nominal between-group differences in average retinal thickness and macular volume at several assessments. However, none of these 16 parameter-by-visit comparisons remained statistically significant after Holm adjustment for multiple testing. The cardiometabolic group was also older and had longer ultrasound time, while cumulative dissipated energy was numerically higher but did not differ significantly between groups. The adjusted models provided no statistically significant evidence that longitudinal postoperative OCT changes differed according to cardiometabolic status, and no statistically significant time-by-group interaction was detected. Thus, the visit-specific findings should be regarded as exploratory and hypothesis-generating. The absence of statistically significant adjusted effects does not demonstrate equivalent retinal responses.

The attenuation of the nominal between-group differences after multiplicity correction and covariate adjustment indicates that the unadjusted findings should not be interpreted as confirmatory evidence of an independent cardiometabolic effect. Consequently, the present data do not establish cardiometabolic disease as an independent determinant of lower postoperative retinal thickness or macular volume. The possibility of chronic retinal remodeling associated with individual cardiometabolic conditions remains biologically plausible but should be regarded as hypothesis-generating and requires confirmation in larger, appropriately matched or prospectively designed studies.

The direction of the nominal structural differences observed in the exploratory analyses may be considered in relation to the pathophysiological mechanisms underlying cardiometabolic disease. Cardiometabolic disease is associated with endothelial dysfunction, chronic low-grade inflammation, oxidative stress, and impaired microvascular autoregulation, all of which affect both the large vessels and the systemic and retinal microcirculation. Prolonged exposure to these factors promotes microvascular remodeling, reduced tissue perfusion, and progressive neurodegenerative retinal changes that can be detected by optical coherence tomography before the onset of clinically apparent manifestations. In this context, the direction of the nominal retinal-thickness and macular-volume differences could be compatible with pre-existing chronic structural alterations; however, the present results do not establish such an association because none of the visit-specific comparisons remained statistically significant after Holm adjustment. This interpretation is supported by recent evidence describing the retina as an accessible window to the systemic microcirculation and demonstrating that OCT-derived structural alterations represent biomarkers of systemic cardiovascular and metabolic disease [5,6,7,8,38,39,40,41].

Our findings are consistent with previous studies reporting structural retinal alterations in patients with cardiometabolic risk factors. Huru et al. [9] reported that metabolic syndrome is associated with changes in macular structures and retinal vascular caliber, suggesting that microvascular impairment and neuroretinal remodeling develop early in the course of systemic disease. Similarly, Aydin et al. demonstrated reduced macular and choroidal thickness in patients with cardiovascular risk factors, supporting the hypothesis that OCT-detected structural alterations reflect the cumulative effects of chronic microvascular dysfunction [9,13,42,43]. The direction of the nominal differences in our exploratory analyses was consistent with these previous reports. However, none of the visit-specific comparisons remained statistically significant after Holm adjustment, and cardiometabolic status was not independently associated with postoperative OCT parameters in the adjusted models. Therefore, the present results do not provide confirmatory evidence of a cardiometabolic structural phenotype. The absence of a statistically significant time-by-group interaction also does not prove identical postoperative responses, and clinically relevant differences cannot be excluded without a prespecified equivalence margin.

Although patients with cardiometabolic disease had longer ultrasound time and numerically higher cumulative dissipated energy, with the CDE difference not reaching statistical significance, the adjusted mixed-effects models did not detect statistically significant differences in longitudinal OCT changes according to cardiometabolic status. Because postoperative inflammation is considered an important mechanism of macular change following cataract surgery, and greater surgical energy or longer surgical times may increase retinal thickening, these variables were included as covariates in the adjusted analyses [3,4,34,35,36,42]. The estimated between-group differences in change from baseline to day 30 were small, but their confidence intervals included both zero and potentially larger positive or negative differences. Accordingly, the results do not establish equivalence or exclude clinically relevant differences. Although the nominal between-group differences in average retinal thickness and macular volume were small, their clinical relevance should be interpreted with caution. Previous studies have demonstrated that spectral-domain OCT provides highly repeatable and reproducible retinal thickness measurements, enabling the detection of subtle structural changes over time. Nevertheless, measurements obtained from different OCT platforms may vary because of differences in segmentation algorithms and acquisition protocols. In the present study, the nominal structural differences were not accompanied by differences in postoperative visual acuity, did not remain statistically significant after Holm adjustment for multiple comparisons, and were not supported by the adjusted longitudinal mixed-effects models. Therefore, although these findings may represent subtle structural variation, their clinical significance remains uncertain and requires confirmation in larger, prospectively designed studies with longer follow-up [44,45,46,47].

From a clinical perspective, unadjusted analyses identified differences in retinal structure between patients with and without cardiometabolic disease. However, cardiometabolic status was not independently associated with postoperative OCT parameters after adjustment for baseline retinal structure and relevant demographic and surgical factors. The analyses also did not detect a statistically significant difference in the longitudinal OCT response between groups. Nevertheless, because equivalence was not formally assessed and follow-up was limited to 30 days, these findings should not be used alone to justify identical monitoring strategies. Postoperative surveillance should remain individualized according to ocular findings, specific systemic diseases, and the patient’s overall clinical risk.

No a priori sample-size calculation was performed because of the retrospective design and fixed number of eligible patients. The unequal group sizes and variability of the change measurements limited precision, particularly for minimum foveal thickness. The calculated minimum detectable differences indicate that smaller potentially clinically relevant effects may have remained undetected. Consequently, the absence of statistically significant between-group differences should be interpreted together with the reported confidence intervals rather than as evidence that no effect exists.

Several study limitations should be considered when interpreting these findings. The cardiometabolic group was clinically heterogeneous, with substantial overlap among hypertension, diabetes mellitus, dyslipidemia, ischemic heart disease, heart failure, and atrial fibrillation. Exploratory sensitivity analyses did not provide multiplicity-adjusted evidence of different postoperative OCT trajectories according to diabetes status or between hypertension alone and other cardiometabolic profiles. Nevertheless, the nominal time-by-diabetes interaction observed for average retinal thickness warrants cautious investigation in a larger study. The small sizes of several diagnostic subgroups and the coexistence of multiple conditions prevented reliable estimation of the independent effect of each disease. Residual confounding related to disease severity, duration, treatment, glycemic and blood-pressure control, and other unmeasured vascular risk factors therefore remains possible.

Additional study limitations should also be considered. First, it was conducted at a single center and included a relatively small number of patients, which may limit the generalizability of the findings. Second, postoperative follow-up was limited to 30 days, precluding the assessment of long-term retinal structural changes. In addition, the cardiometabolic disease group comprised patients with different conditions and combinations of cardiovascular risk factors, without separate analyses of the impact of each condition on OCT parameters. Finally, no equivalence margin was prespecified, and the study was not designed or powered as an equivalence study; therefore, nonsignificant between-group findings should not be interpreted as evidence of equivalent responses. Nevertheless, the use of a standardized surgical protocol, serial OCT assessments, and the inclusion of a control group enabled a direct and robust comparison of retinal structural changes associated with cardiometabolic disease following uncomplicated phacoemulsification.

## 5. Conclusions

All evaluated macular OCT parameters increased during the first postoperative month following uncomplicated phacoemulsification. After adjustment for baseline OCT values, age, sex, cumulative dissipated energy, and ultrasound time, no statistically significant association was detected between cardiometabolic status and the temporal evolution of the postoperative OCT parameters. However, these findings do not demonstrate equivalence, and clinically relevant differences cannot be definitively excluded because no equivalence margin was prespecified. Within these limitations, cardiometabolic status alone was not identified as an independent predictor of the early postoperative structural retinal response. Postoperative surveillance should remain individualized according to ocular findings, specific systemic diseases, and the patient’s overall clinical risk.

## Figures and Tables

**Table 1:** Baseline demographic, clinical characteristics and comorbidities of the study population.

Variable	Category	n	%
Age, years	Median (IQR)	71.0 (11.0)	
Sex	Female	64	57.7
	Male	47	42.3
Residence	Rural	26	23.4
	Urban	85	76.6
Operated eye	Right eye (RE)	61	55.0
	Left eye (LE)	50	45.0
Comorbidities	Present	70	63.1
	Absent	41	36.9
	Hypertension	67	60.4
	Type 2 diabetes mellitus	25	22.5
	Dyslipidemia	19	17.1
	Ischemic heart disease	13	11.7
	Heart failure	5	4.5
	Atrial fibrillation	2	1.8

Cardiometabolic pathology was defined as the presence of at least one of the following: hypertension, type 2 diabetes mellitus, dyslipidemia, ischemic heart disease, heart failure, or atrial fibrillation.

**Table 2:** Preoperative and postoperative visual acuity (decimal notation) in the study population.

Variable	Median (IQR)	Median Difference (95% CI)	*p*
Preoperative visual acuity	0.300 (0.400)		
Postoperative visual acuity (day 30)	1.00 (0.100)	0.550 (0.500 to 0.600)	<0.001 *

* Wilcoxon signed-rank test for paired observations. The effect estimate represents the median postoperative improvement from the preoperative assessment. Visual acuity is expressed in decimal notation. IQR, interquartile range; CI, confidence interval.

**Table 3:** Intraoperative parameters of phacoemulsification (N = 111).

Variable	Median (IQR)	Shapiro–Wilk *p*
Cumulative dissipated energy (CDE), %·s	6.26 (7.99)	<0.001
Average phaco power, %	27.1 (13.6)	0.011
Ultrasound time, s	45 (38.0)	0.010
Torsional time, s	43 (39.0)	0.030
Aspiration time, s	259 (116)	<0.001
Estimated fluid used, mL	79 (26.5)	<0.001

All procedures were performed using the Infiniti Vision System with OZil torsional handpiece (Alcon Laboratories, Fort Worth, TX, USA). IQR = interquartile range. Normality was assessed using the Shapiro–Wilk test.

**Table 4:** Longitudinal changes in macular OCT parameters (N = 111).

Parameter	Preoperative	Day 1	Day 7	Day 30	χ^2^	*p*	Kendall’s W
Minimum foveal thickness (μm)	201 (30.0)	202 (32.0)	206 (35.0)	210 (33.0)	65.8	<0.001	0.198
Central sector thickness (μm)	246 (31.0)	245 (31.0)	250 (32.5)	253 (35.0)	116	<0.001	0.348
Average retinal thickness (μm)	273 (22.0)	272 (20.5)	277 (21.0)	280 (20.0)	132	<0.001	0.396
Macular volume (mm^3^)	7.74 (0.660)	7.70 (0.625)	7.81 (0.605)	7.90 (0.595)	142	<0.001	0.426

Data are presented as median (interquartile range). Friedman test, df = 3. Kendall’s W was calculated as the Friedman chi-square statistic divided by $N\left(k-1\right)$, where N=111 and k=4 assessments.

**Table 5:** Holm-adjusted *p*-values for Durbin–Conover pairwise comparisons of macular OCT parameters.

Comparison	Minimum Foveal Thickness	Central Sector Thickness	Average Retinal Thickness	Macular Volume
Preoperative vs. Day 1	0.365	0.185	0.020	0.008
Preoperative vs. Day 7	<0.001	<0.001	<0.001	<0.001
Preoperative vs. Day 30	<0.001	<0.001	<0.001	<0.001
Day 1 vs. Day 7	<0.001	<0.001	<0.001	<0.001
Day 1 vs. Day 30	<0.001	<0.001	<0.001	<0.001
Day 7 vs. Day 30	<0.001	<0.001	<0.001	<0.001

Values are *p*-values adjusted using the Holm procedure across the six pairwise comparisons performed separately for each OCT parameter.

**Table 6:** Baseline characteristics of patients with and without cardiometabolic pathology.

Variable	Without Pathology (n = 41)	With Pathology (n = 70)	Rank-Biserial Correlation (95% CI)	*p*
Age, years	68.0 (13.0)	73.0 (11.75)	−0.420 (−0.613 to −0.226)	<0.001
Preoperative visual acuity	0.300 (0.500)	0.300 (0.300)	0.002 (−0.230 to 0.234)	0.988
Postoperative visual acuity	0.900 (0.100)	1.000 (0.100)	−0.088 (−0.287 to 0.112)	0.392
Cumulative dissipated energy (%·s)	5.19 (5.99)	6.50 (7.67)	−0.216 (−0.435 to 0.003)	0.059
Ultrasound time (s)	39.0 (35.0)	47.5 (32.75)	−0.232 (−0.451 to −0.012)	0.042
Aspiration time (s)	251 (79.0)	261 (124.5)	−0.039 (−0.257 to 0.180)	0.737
Estimated fluid used (mL)	75.0 (24.0)	79.0 (27.75)	−0.118 (−0.335 to 0.098)	0.300

Data are median (interquartile range). Effect estimates are rank-biserial correlations calculated as without cardiometabolic disease versus with cardiometabolic disease. Negative values indicate that observations tend to be higher in the cardiometabolic group. Confidence intervals were calculated using the large-sample variance of the pairwise comparison probabilities, with ties assigned half weight. *p*-values were obtained using two-sided Mann–Whitney U tests. CI, confidence interval.

**Table 7:** Exploratory visit-specific comparisons of all macular OCT parameters.

Parameter	Timepoint	Without Cardiometabolic Disease (n = 41)	With Cardiometabolic Disease (n = 70)	Effect Estimate (95% CI)	Test	Nominal *p*	Holm-Adjusted *p*
Minimum foveal thickness (µm)	Preoperative	201.2 ± 23.1	207.0 ± 23.6	−5.80 (−14.92 to 3.31)	*t* test	0.2097	1.0000
	Day 1	201.2 ± 23.3	206.6 ± 23.7	−5.38 (−14.57 to 3.81)	*t* test	0.2486	1.0000
	Day 7	205.3 ± 25.4	209.9 ± 26.4	−4.67 (−14.81 to 5.46)	*t* test	0.3628	1.0000
	Day 30	207.8 ± 24.3	213.6 ± 28.1	−5.74 (−16.18 to 4.70)	*t* test	0.2781	1.0000
Central sector thickness (µm)	Preoperative	241.1 ± 22.4	246.1 ± 21.9	−5.05 (−13.65 to 3.56)	*t* test	0.2476	1.0000
	Day 1	240.9 ± 23.5	246.4 ± 21.6	−5.48 (−14.19 to 3.22)	*t* test	0.2146	1.0000
	Day 7	245.2 ± 24.1	250.7 ± 23.1	−5.50 (−14.65 to 3.65)	*t* test	0.2361	1.0000
	Day 30	248.4 ± 24.2	254.5 ± 25.0	−6.05 (−15.69 to 3.60)	*t* test	0.2166	1.0000
Average retinal thickness (µm)	Preoperative	278.2 ± 13.8	271.6 ± 16.2	6.57 (0.57 to 12.57)	*t* test	0.0323	0.3875
	Day 1	278.0 ± 13.1	270.8 ± 15.3	7.24 (1.59 to 12.89)	*t* test	0.0125	0.1746
	Day 7	282.3 ± 13.2	275.1 ± 16.1	7.20 (1.32 to 13.07)	*t* test	0.0168	0.2181
	Day 30	285 (20.0)	277 (21.0)	0.299 (0.088 to 0.509)	Mann–Whitney	0.0089	0.1333
Macular volume (mm^3^)	Preoperative	7.868 ± 0.388	7.699 ± 0.462	0.169 (−0.001 to 0.339)	*t* test	0.0516	0.4648
	Day 1	7.843 ± 0.371	7.669 ± 0.443	0.174 (0.011 to 0.337)	*t* test	0.0365	0.4015
	Day 7	7.954 ± 0.384	7.779 ± 0.447	0.174 (0.009 to 0.340)	*t* test	0.0393	0.4015
	Day 30	8.03 (0.600)	7.81 (0.598)	0.302 (0.092 to 0.511)	Mann–Whitney	0.0082	0.1315

Values are mean ± standard deviation for normally distributed rows and median (interquartile range) for rows with non-normality in either group. Effect estimates are calculated as without cardiometabolic disease minus or relative to with cardiometabolic disease. For *t*-test rows, estimates are mean differences with t-based 95% confidence intervals. For Mann–Whitney rows, estimates are rank-biserial correlations with large-sample 95% confidence intervals based on pairwise comparison probabilities. Positive rank-biserial correlations indicate that observations tend to be higher in patients without cardiometabolic disease. Nominal *p*-values are unadjusted; Holm-adjusted *p*-values control the family-wise error rate across the 16 parameter-by-visit comparisons. CI = confidence interval.

**Table 8:** Secondary descriptive comparison of changes from baseline to postoperative day 30.

Parameter	Without Pathology (n = 41)	With Pathology (n = 70)	Between Group Difference (95% CI)	*p*
Δ Minimum foveal thickness (μm)	+6.63 ± 8.45	+6.57 ± 13.66	−0.06 (−4.74 to 4.62)	0.979
Δ Central sector thickness (μm)	+7.34 ± 7.65	+8.34 ± 11.41	1.00 (−2.97 to 4.97)	0.618
Δ Average retinal thickness (μm)	+6.68 ± 6.65	+5.57 ± 6.76	−1.11 (−3.73 to 1.51)	0.402
Δ Macular volume (mm^3^)	+0.178 ± 0.179	+0.141 ± 0.164	−0.036 (−0.102 to 0.030)	0.279

Data are presented as mean ± SD. Between-group differences are calculated as the change in patients with cardiometabolic disease minus the change in patients without cardiometabolic disease. CI = confidence interval; Δ = change from the preoperative assessment to day 30. *p*-values were obtained using independent-samples *t*-tests. Change-score comparisons were secondary and unadjusted. Primary inference was based on the longitudinal mixed-effects models, including sensitivity analyses of postoperative measurements adjusted for the corresponding baseline OCT value.

**Table 9:** Adjusted linear mixed-effects models for longitudinal OCT measurements.

Outcome	Day 1 vs. Baseline	Day 7 vs. Baseline	Day 30 vs. Baseline	Global Interaction χ^2^ (df = 3)	*p*
Minimum foveal thickness, µm	−0.42 (−4.44 to 3.59)	−1.13 (−5.14 to 2.88)	−0.06 (−4.08 to 3.95)	0.385	0.943
Central sector thickness, µm	0.44 (−2.76 to 3.63)	0.45 (−2.74 to 3.65)	1.00 (−2.19 to 4.20)	0.380	0.944
Average retinal thickness, µm	−0.68 (−2.93 to 1.58)	−0.63 (−2.89 to 1.63)	−1.11 (−3.37 to 1.15)	0.946	0.814
Macular volume, mm^3^	−0.005 (−0.061 to 0.050)	−0.006 (−0.061 to 0.050)	−0.036 (−0.092 to 0.019)	2.052	0.562

Estimates are time-by-cardiometabolic-status interaction contrasts with 95% confidence intervals and represent the adjusted difference between groups in change from the preoperative assessment. Positive estimates indicate a greater increase from baseline in patients with cardiometabolic disease. Models included a patient-specific random intercept and fixed effects for assessment time, cardiometabolic status, their interaction, age, sex, cumulative dissipated energy, and ultrasound time. Global interactions were evaluated using likelihood-ratio tests.

## Data Availability

The data presented in this study are available upon request from the corresponding authors.
